# Supplementary material for: Structural basis for SARS-CoV-2 Delta variant recognition of ACE2 receptor and broadly neutralizing antibodies
Source: Nat Commun. 2022 Feb 15;13:871. doi: 10.1038/s41467-022-28528-w (PMC8847413; doi:10.1038/s41467-022-28528-w)
Supplement: Supplementary file 1 — Supplementary information [file 41467_2022_28528_MOESM1_ESM.pdf]

Supplementary information

Structural basis for SARS-CoV-2 Delta variant recognition of ACE2 receptor and broadly neutralizing antibodies

Yifan Wang[1,2,#], Caixuan Liu[1,2,#], Chao Zhang[3,#], Yanxing Wang[1,#], Qin Hong[1,2,#], Shiqi Xu[3], Zuyang Li[1,2], Yong Yang[3], Zhong Huang[3,*], Yao Cong[1,2,*]

[1] State Key Laboratory of Molecular Biology, National Center for Protein Science Shanghai, Shanghai Institute of Biochemistry and Cell Biology, Center for Excellence in Molecular Cell Science, Chinese Academy of Sciences, Shanghai 200031, China.

[2] University of Chinese Academy of Sciences, Beijing 100049, China.

[3] CAS Key Laboratory of Molecular Virology and Immunology, Institut Pasteur of Shanghai, Chinese Academy of Sciences, University of Chinese Academy of Sciences, Shanghai 200031, China.

[#] These authors contributed equally to this work.

*To whom correspondence may be addressed. Email: cong@sibcb.ac.cn, huangzhong@ips.ac.cn.

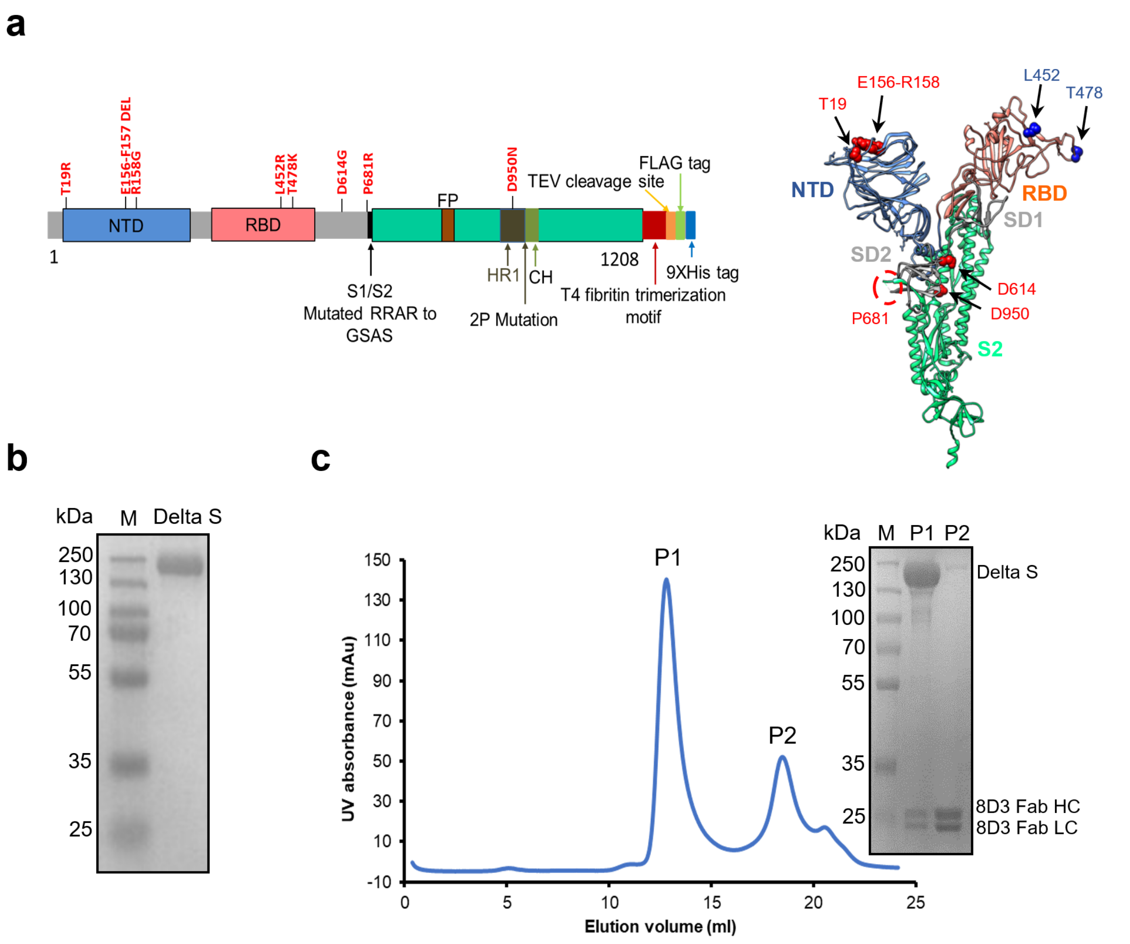

Supplementary Fig. 1 Purification of Delta variant S and S-8D3 Fab complex. a Schematic diagram of the Delta variant S organization in this study (left, positions of all mutations are shown in red), and the model of a SARS-CoV-2 S protomer (right) with mutation sites of the Delta variant shown as sphere. **b** SDS-PAGE analysis of the purified Delta variant S protein. Lane M, protein marker. Representative images of two independent experiments are shown. **c** Size-exclusion chromatogram and SDS-PAGE analysis of the formed Delta S-8D3 Fab complex. Lane M, protein marker. Representative images of two independent experiments are shown. Source data are provided as a Source Data file.

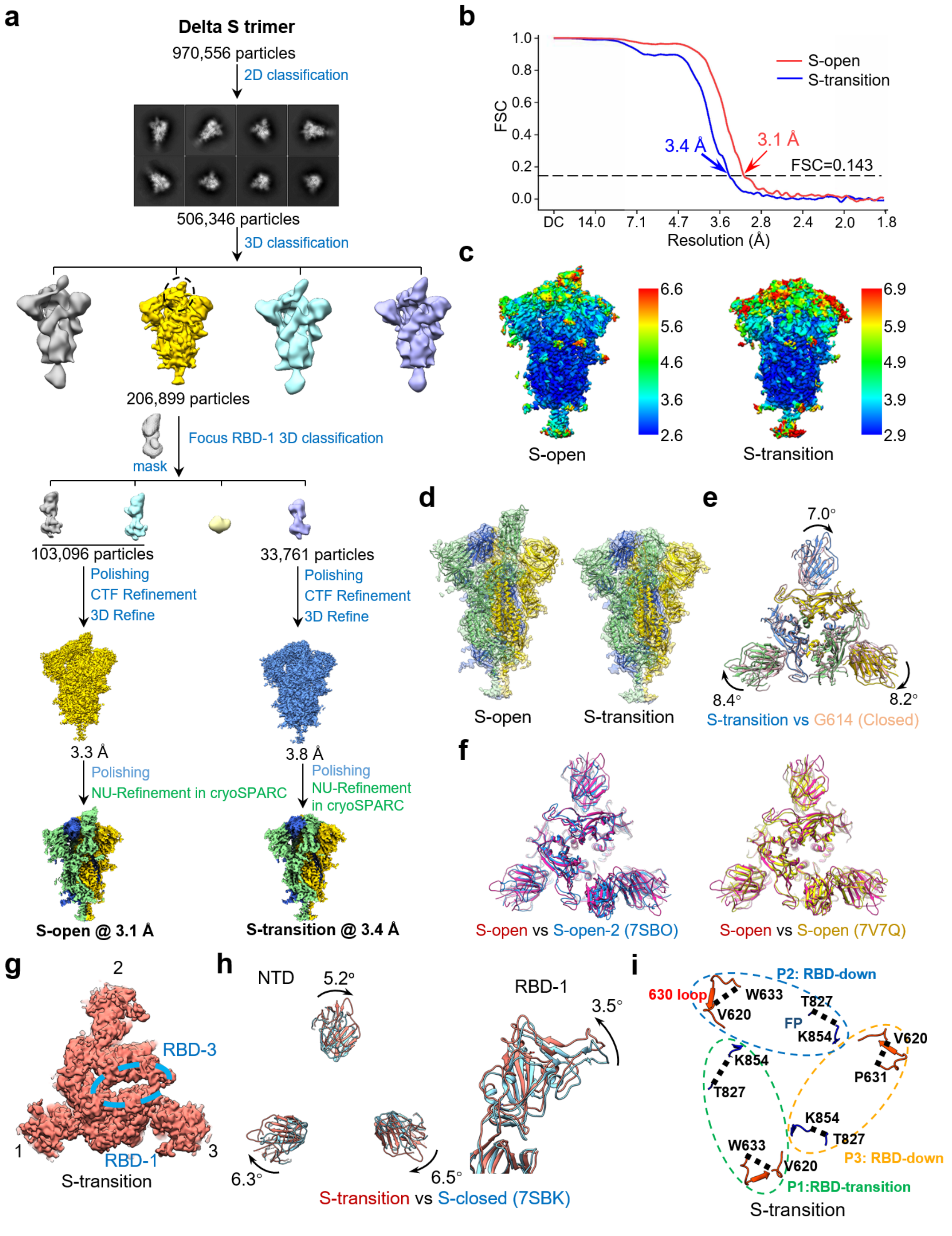

Supplementary Fig. 2 Cryo-EM analysis on the Delta variant S trimer. a Data processing workflow for structure determination of the Delta variant S trimer. The reference-free 2D class averages are also presented. **b** Resolution assessment of Delta S-open and S-transition maps by FSC at 0.143 criterion. **c** Local resolution evaluation of the Delta S-open and S-transition maps. **d** Model-map fitting of the Delta S-open and S-transition structures. **e** Top view of overlaid Delta S-transition (in color) and G614

S-close (PDB: 7KRQ, pink) structures. **f** Overlaid structural comparison between our Delta S-open state with Delta S-open2 (7SBO[54], dodger blue, left) and S-open (7V7Q[57], gold, right) state. **g** Top view of our Delta S-transition cryo-EM map, which appears asymmetric with a gap emerging between RBD-1 and -3 (indicated by dotted blue oval), which is not the case between RBD-2 and -3. **h** Overlaid structural comparison between our Delta S-transition state with Delta S-closed (7SBK[54]) state, indicating a clockwise rotation/untwist of our Delta S-transition relative to the Delta S-closed (left). Side view of the overlaid RBD-1 between the two structures (right), showing that the RBD-1 of our Delta S-transition is slightly lifted for 3.5° relative that of their Delta S-closed. **i** In Delta S-transition, all three FPs (blue) are disordered, all the 630 loops (red) are partially disordered.

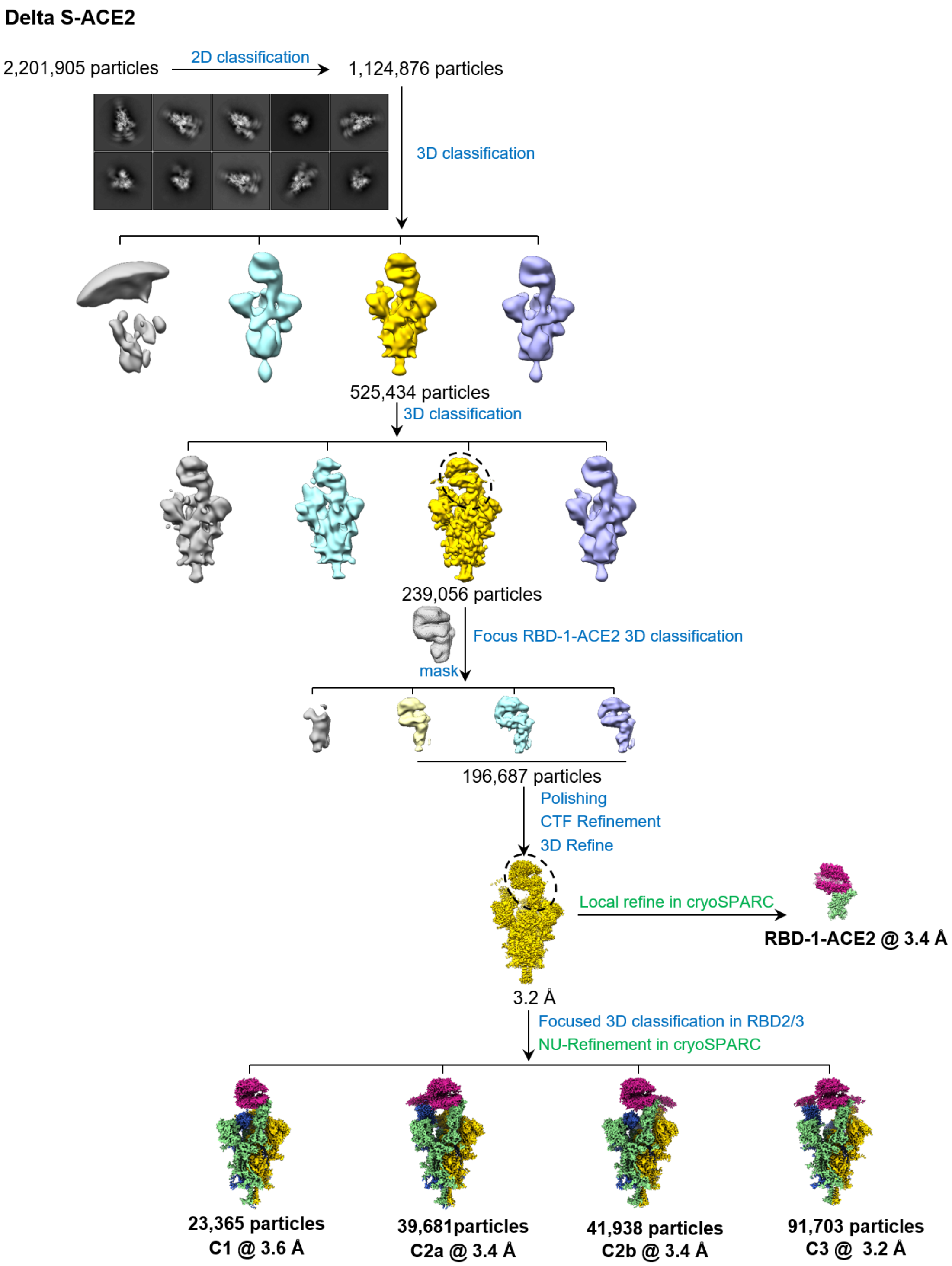

Supplementary Fig. 3 Cryo-EM data processing procedure for the Delta S-ACE2 complex.

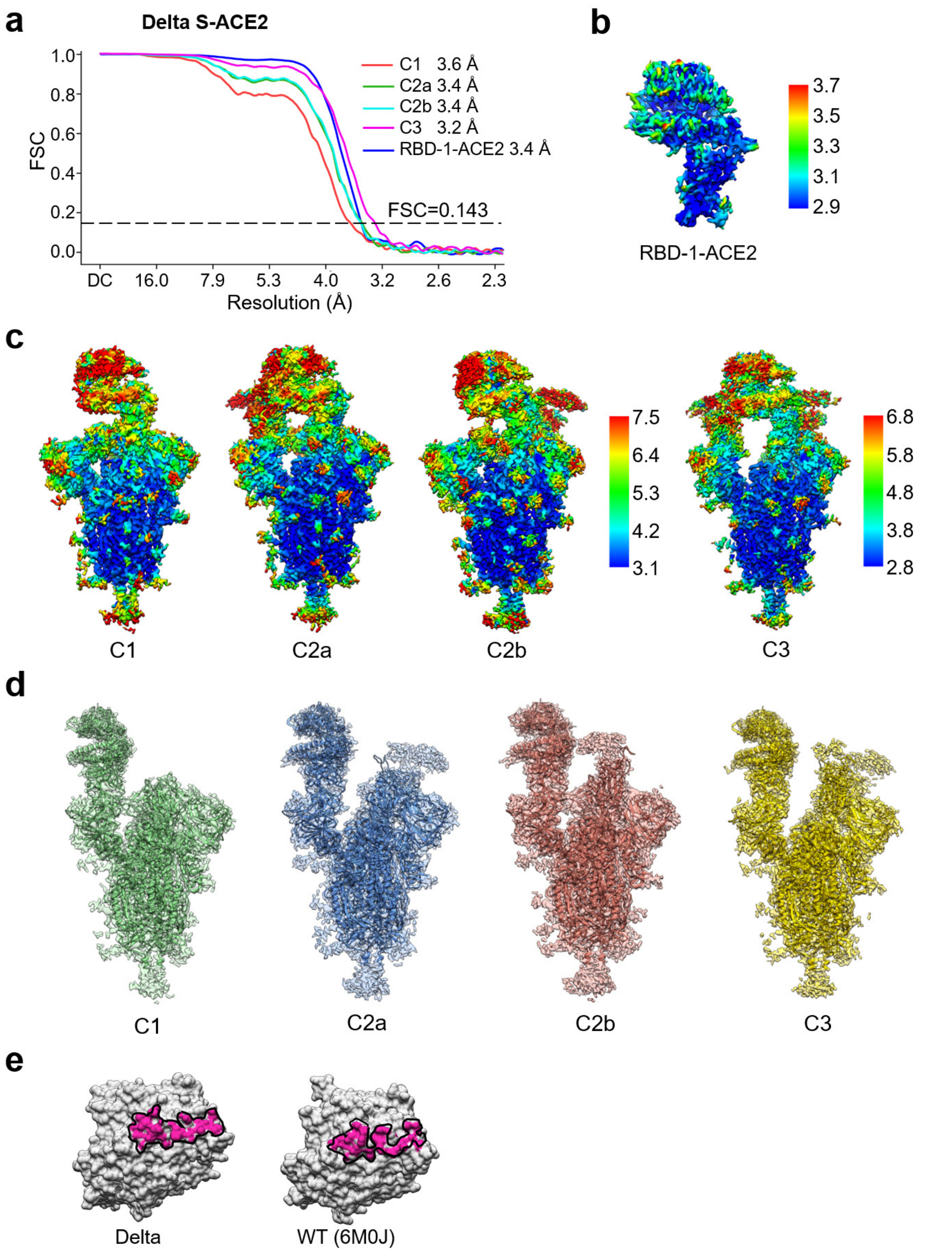

Supplementary Fig. 4 Cryo-EM analysis on the Delta S-ACE2 complex. a Resolution assessment of the cryo-EM maps by FSC at 0.143 criterion. **b-c** Local resolution evaluation of the RBD-1-ACE2 map (b) and the Delta S-ACE2 complex maps (c). **d** Model-map fitting for the four states of the Delta S-ACE2 complex. **e** The footprint (in violet red) for the Delta RBD (left) and WT RBD (6M0J[5],right) interactions on the ACE2 surface, with residues in proximity to RBD-1 (< 4 Å) indicated.

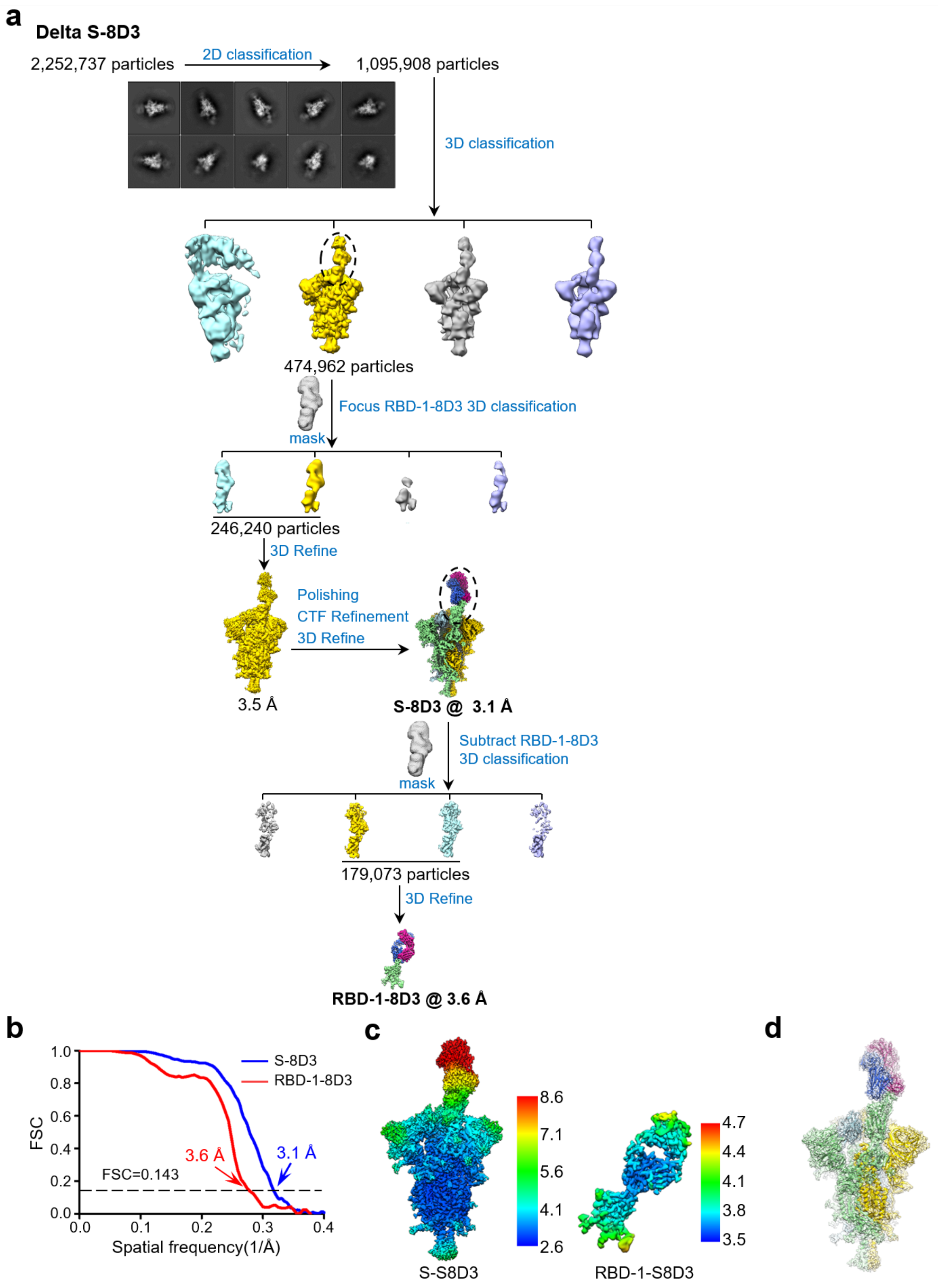

Supplementary Fig. 5 Cryo-EM analysis on the Delta variant S-8D3 Fab complex. **a** Data processing workflow for the Delta variant S-8D3 Fab complex. The reference-free 2D class averages are also presented. **b** Resolution assessment of the cryo-EM maps by FSC at 0.143 criterion. **c** Local resolution evaluation of the Delta S-8D3 and RBD-1-8D3 maps. **d** Model-map fitting for the Delta S-8D3 complex.

Supplementary Table 1. Cryo-EM data collection and refinement statistics for Delta S, Delta S-ACE2, and Delta S-8D3

	Delta S		Delta S-ACE2					Delta S-8D3	
**Data collection**									
EM equipment	Titan Krios		Titan Krios					Titan Krios	
Voltage (kV)	300		300					300	
Detector	Gatan K3 camera		Gatan K3 camera					Gatan K3 camera	
Pixel size (Å)	0.893		1.093					1.093	
Electron dose (e^-/Å^2)	50.2		50.2					50.2	
Exposure time (s)	2		3					3	
Frames	40		30					30	
Defocus range (μm)	-0.8 to -2.5		-0.8 to -2.5					-0.8 to -2.5	
**Reconstruction**									
Softwares	Relion 3.1&cryoSPARC								
Structures	S-open	S-transition	C1	C2a	C2b	C3	RBD-1-ACE2	S-8D3	RBD-1-8D3
Final particles	103,096	33,761	23,365	39,681	41,938	91,703	196,687	246,240	179,073
Symmetry	C1	C1	C1	C1	C1	C1	C1	C1	C1
FSC threshold	0.143								
Final overall resolution (Å)	3.1	3.4	3.6	3.4	3.4	3.2	3.4	3.1	3.6
Resolution Range (Å)	2.6-6.6	2.9-6.9	3.1-7.5	3.1-7.5	3.1-7.5	2.8-6.8	2.9-3.7	2.6-8.6	3.5-4.7
**Atomic modeling**									
Softwares	Rosetta & Phenix & Coot								
Initial model ID	7DK3	7KRS	7DF4	7DF4	7DF4	7DF4	7DF4	7DK3; 5H2B	7DK3; 5H2B
Averaged Bfactor	88.9	112.3	183.8	144.8	150.8	71.4	43.7	89.3	82.6
number of non-H atoms, residues and ligands	25,074; 3,206; 0	25,085; 3,206; 0	29,741; 3,777; 0	29,741; 3,777; 0	29,741; 3,777; 0	29,741; 3,777; 0	6,413; 791; 0	28,186; 3,611; 0	4,858; 625; 0
Rms deviations									
Bond length (Å)	0.0066	0.0042	0.0043	0.0045	0.0045	0.0048	0.0053	0.0046	0.0044
Bond Angle (°)	1.09	1.06	0.98	0.99	0.99	1.01	1.12	1.01	1.10
Ramachandran plot (%)									
Favored	95.85	95.76	97.40	96.95	97.16	97.21	95.17	96.07	96.28
Allowed	4.15	4.24	2.57	2.93	2.81	2.79	4.83	3.93	3.72
Outliers	0.00	0.00	0.03	0.12	0.03	0.00	0.00	0.00	0.00
Molprobity score	1.45	1.46	1.02	1.10	1.08	1.08	1.51	1.37	1.38
Clash score	3.69	3.81	1.60	1.65	1.76	1.76	3.91	3.14	3.37

Supplementary Table 2. Delta RBD-1-ACE2 structure revealed RBD-1/ACE2 interactions

Delta S RBD-1		ACE2		Interaction	Distance(Å)
Residue	Atom	Residue	Atom		
LYS 417	[NZ]	ASP 30	[OD2]	Salt bridge	3.56
ASN 487	[ND2]	GLN 24	[OE1]	H bond	2.42
TYR 489	[OH]	TYR 83	[OH]	H bond	3.11
THR 500	[OG1]	TYR 41	[OH]	H bond	2.51
GLY 502	[N]	LYS 353	[O]	H bond	3.11
TYR 505	[OH]	GLU 37	[OE2]	H bond	2.93
TYR 449	[OH]	GLN 42	[NE2]	H bond	2.42
ASN 487	[OD1]	TYR 83	[OH]	H bond	2.66
PHE 490	[O]	LYS 31	[NZ]	H bond	3.24
THR 500	[O]	TYR 41	[OH]	H bond	3.30
TYR 505	[OH]	ARG 393	[NH2]	H bond	3.75

Supplementary Table 3. Contacting residues (a sidechain distance cut off 4 Å) at the Delta RBD-1/ACE2 interface

Delta S RBD-1	ACE2
K417	D30
Y449	Q42
Y453	H34
L455	D30, H34
F456	T27, D30, K31
Y473	T27
A475	Q24, T27
G476	Q24
F486	M82, Y83
N487	Q24, Y83
Y489	T27, F28, K31, Y83
F490	K31
Q493	K31, H34
G496	D38, K353
Q498	Y41, L45
T500	Y41, D355, R357
N501	Y41, K353
G502	K353, G354
Y505	E37, K353, R393

Supplementary Table 4. Delta S RBD-1-8D3 structure revealed RBD-1/8D3 interactions

Delta S RBD-1		8D3		Interaction	Distance(Å)
Residue	Atom	Residue	Atom		
TYR 473	[OH]	GLU 31	[OE1]	H bond	3.76
SER 477	[N]	ASP 99	[OD2]	H bond	3.34
SER 477	[OG]	ASP 99	[OD1]	H bond	2.54
ASN 487	[ND2]	TYR 35	[OH]	H bond	3.11
ASN 487	[OD1]	THR 33	[OG1]	H bond	3.64
TYR 489	[OH]	ASN 52	[ND2]	H bond	2.43
LYS 478	[NZ]	ASN 92	[O]	H bond	3.38
ASN 487	[ND2]	TYR 94	[OH]	H bond	3.09
SER 477	[O]	ASN 32	[ND2]	H bond	3.80

Heavy chain

Light chain

Supplementary Table 5. Contacting residues (a sidechain distance cut off 4 Å) at the Delta RBD-1/8D3 interface

Delta S RBD-1	8D3
F456	N54, I55
Y473	E31
Q474	Y101
A475	Y101
G476	D99, Y101
S477	D99, Y101, N32, Y91
K478	Y91, N92, Y94
P479	Y91
G485	D57
F486	S59, D57, G50, Y94
N487	T33, Y35, D99, Y94
Y489	N52, D57

Heavy chain

Light chain
