# Supplementary material for: Structural basis for SARS-CoV-2 Delta variant recognition of ACE2 receptor and broadly neutralizing antibodies
Source: Nat Commun. 2022 Feb 15;13:871. doi: 10.1038/s41467-022-28528-w (PMC8847413; doi:10.1038/s41467-022-28528-w)
Supplement: Supplementary file 3 — Description of Additional Supplementary Files [file 41467_2022_28528_MOESM3_ESM.pdf]

Description of Additional Supplementary Files

File Name: Supplementary Movie 1
Description: Motion mode 1 revealed by 3DVA of the Delta S-open dataset.

File Name: Supplementary Movie 2
Description: Motion mode 2 revealed by 3DVA of the Delta S-open dataset.

File Name: Supplementary Movie 3
Description: 3DVA motion mode 1 of the Delta S-ACE2 complex.

File Name: Supplementary Movie 4
Description: 3DVA motion mode 2 of the Delta S-ACE2 complex.

File Name: Supplementary Movie 5
Description: 3DVA motion mode 3 of the Delta S-ACE2 complex.
