# Supplementary material for: Structural basis for SARS-CoV-2 Delta variant recognition of ACE2 receptor and broadly neutralizing antibodies
Source: Nat Commun. 2022 Feb 15;13:871. doi: 10.1038/s41467-022-28528-w (PMC8847413; doi:10.1038/s41467-022-28528-w)
Supplement: Supplementary file 9 — Reporting Summary [file 41467_2022_28528_MOESM9_ESM.pdf]

nature portfolio

Corresponding author(s): Yao Cong

Last updated by author(s): Jan 18, 2022

Reporting Summary

Nature Portfolio wishes to improve the reproducibility of the work that we publish. This form provides structure for consistency and transparency in reporting. For further information on Nature Portfolio policies, see our Editorial Policies and the Editorial Policy Checklist.

Statistics

For all statistical analyses, confirm that the following items are present in the figure legend, table legend, main text, or Methods section.

n/a	Confirmed	
☐	☒	The exact sample size (*n*) for each experimental group/condition, given as a discrete number and unit of measurement
☐	☒	A statement on whether measurements were taken from distinct samples or whether the same sample was measured repeatedly
☒	☐	The statistical test(s) used AND whether they are one- or two-sided<br>*Only common tests should be described solely by name; describe more complex techniques in the Methods section.*
☒	☐	A description of all covariates tested
☒	☐	A description of any assumptions or corrections, such as tests of normality and adjustment for multiple comparisons
☐	☒	A full description of the statistical parameters including central tendency (e.g. means) or other basic estimates (e.g. regression coefficient) AND variation (e.g. standard deviation) or associated estimates of uncertainty (e.g. confidence intervals)
☒	☐	For null hypothesis testing, the test statistic (e.g. *F*, *t*, *r*) with confidence intervals, effect sizes, degrees of freedom and *P* value noted<br>*Give P values as exact values whenever suitable.*
☒	☐	For Bayesian analysis, information on the choice of priors and Markov chain Monte Carlo settings
☒	☐	For hierarchical and complex designs, identification of the appropriate level for tests and full reporting of outcomes
☒	☐	Estimates of effect sizes (e.g. Cohen's *d*, Pearson's *r*), indicating how they were calculated

Our web collection on statistics for biologists contains articles on many of the points above.

Software and code

Policy information about availability of computer code

Data collection	FEI TEM user interface, EPU 2.11
Data analysis	Octet Data Analysis 11.0, GraphPad Prism (version 8), MotionCor2, RELION 3.1, CTFFIND 4.1.8, Phenix 1.12-2829, Rosetta 2017, COOT 0.8.7, UCSF Chimera 1.10.2, UCSF ChimeraX 1.0, PDBePISA, cryoSPARC v3.2.0, deepEMhancer

For manuscripts utilizing custom algorithms or software that are central to the research but not yet described in published literature, software must be made available to editors and reviewers. We strongly encourage code deposition in a community repository (e.g. GitHub). See the Nature Portfolio guidelines for submitting code & software for further information.

Data

Policy information about availability of data

All manuscripts must include a data availability statement. This statement should provide the following information, where applicable:

- Accession codes, unique identifiers, or web links for publicly available datasets
- A description of any restrictions on data availability
- For clinical datasets or third party data, please ensure that the statement adheres to our policy

All data presented in this study are available within the figures and in the Supplementary Information. For the SARS-CoV-2 Delta variant, related cryo-EM maps have been deposited at the Electron Microscopy Data Bank with accession codes EMD-32359 [https://www.ebi.ac.uk/pdbe/entry/emdb/EMD-32359], EMD-32360 [https://www.ebi.ac.uk/pdbe/entry/emdb/EMD-32360], EMD-32361 [https://www.ebi.ac.uk/pdbe/entry/emdb/EMD-32361], EMD-32362 [https://www.ebi.ac.uk/pdbe/entry/emdb/EMD-32362], EMD-32363 [https://www.ebi.ac.uk/pdbe/entry/emdb/EMD-32363], EMD-32364 [https://www.ebi.ac.uk/pdbe/entry/emdb/EMD-32364], EMD-32365 [https://www.ebi.ac.uk/pdbe/entry/emdb/EMD-32365], EMD-32366 [https://www.ebi.ac.uk/pdbe/entry/emdb/EMD-32366], and EMD-32367 [https://www.ebi.ac.uk/pdbe/entry/emdb/EMD-32367], and associated atomic models have been deposited in the Protein Data Bank with accession

codes 7W92 [https://doi.org/10.2210/pdb7W92/pdb], 7W94 [https://doi.org/10.2210/pdb7W94/pdb], 7W98 [https://doi.org/10.2210/pdb7W98/pdb], 7W99 [https://doi.org/10.2210/pdb7W99/pdb], 7W9B [https://doi.org/10.2210/pdb7W9B/pdb], 7W9C [https://doi.org/10.2210/pdb7W9C/pdb], 7W9E [https://doi.org/10.2210/pdb7W9E/pdb], 7W9F [https://doi.org/10.2210/pdb7W9F/pdb] and 7W9I [https://doi.org/10.2210/pdb7W9I/pdb] for S-open, S-transition, S-ACE2-C1, S-ACE2-C2a, S-ACE2-C2b, S-ACE2-C3, S-8D3,RBD-1-8D3, and RBD-1-ACE2, respectively. Source data are provided with this paper.

Field-specific reporting

Please select the one below that is the best fit for your research. If you are not sure, read the appropriate sections before making your selection.

- [x] Life sciences
- [ ] Behavioural & social sciences
- [ ] Ecological, evolutionary & environmental sciences

For a reference copy of the document with all sections, see nature.com/documents/nr-reporting-summary-flat.pdf

Life sciences study design

All studies must disclose on these points even when the disclosure is negative.

Sample size	No statistical methods were used to determine sample size for EM data. Sample sizes were determined based on the ability to get sufficient particles for 3D reconstruction and particles with good features are selected.
Data exclusions	The cryo-EM images of poor quality were deleted based on the defocus, astigmatism and reslution for better resolution.
Replication	Experimental findings were reliably reproduced. Most of the experiments were replicated two or three times.
Randomization	Randomization was not relevant for this study, as data were collected automatically and there were no samples allocated into control and experimental groups.
Blinding	Blinding was not relevant for this study, as data were collected automatically, and the data under investigation need to be known.

Reporting for specific materials, systems and methods

We require information from authors about some types of materials, experimental systems and methods used in many studies. Here, indicate whether each material, system or method listed is relevant to your study. If you are not sure if a list item applies to your research, read the appropriate section before selecting a response.

Materials & experimental systems

n/a	Involved in the study	
[ ]	[x]	Antibodies
[ ]	[x]	Eukaryotic cell lines
[x]	[ ]	Palaeontology and archaeology
[x]	[ ]	Animals and other organisms
[x]	[ ]	Human research participants
[x]	[ ]	Clinical data
[x]	[ ]	Dual use research of concern

Methods

n/a	Involved in the study	
[x]	[ ]	ChIP-seq
[x]	[ ]	Flow cytometry
[x]	[ ]	MRI-based neuroimaging

Antibodies

Antibodies used	HRP-conjugated anti-mouse IgG (Sigma, A0168-1ML); anti-RBD MAbs 8D3, 2G3, 3A2, 2H2, and 3C1 (50 ng/well [1 µg/mL] for each MAb in ELISA) were prepared in Zhong Huang's lab.
Validation	The specifications of commercially available HRP-conjugated anti-mouse IgG can be found on the manufacture's website (https://www.sigmaaldrich.com/HK/zh/product/sigma/a0168). Anti-RBD MAbs 2H2 and 3C1 have been validated previously (Zhang et al. Nature communications. 2021, 12, 264; https://doi.org/10.1038/s41467-020-20465-w).

Eukaryotic cell lines

Policy information about cell lines

Cell line source(s)	Human ACE2-expressing HEK 293T cells (293T-hACE2) were generated in Zhong Huang's lab; HEK293F suspension cells, Thermo fisher.
Authentication	293T-hACE2 cells have been validated previously (Zhang et al. Nature communications. 2021, 12, 264; https://doi.org/10.1038/s41467-020-20465-w). HEK293F cells were not authenticated further after purchase.
Mycoplasma contamination	Cell lines have not recently been tested for Mycoplasma contamination.

Commonly misidentified lines<br>(See ICLAC register)	No commonly misidentified lines were used.
